# Chemical Composition, Antiviral and Antiproliferative Activity of Extracts from *Agrimonia eupatoria* L.

**DOI:** 10.3390/molecules31214019

**Published:** 2026-11-08

**Authors:** Plamena Staleva, Mariana Kamenova-Nacheva, Antoaneta Trendafilova, Zhanina Petkova, Ivan Iliev, Aleksandra Rangelova, Neli Vilhelmova-Ilieva

**Affiliations:** 1 Institute of Organic Chemistry with Centre of Phytochemistry, Bulgarian Academy of Sciences, Acad. G. Bonchev Str., bl. 9, 1113 Sofia, Bulgaria; mariana.nacheva@orgchm.bas.bg (M.K.-N.); antoaneta.trendafilova@orgchm.bas.bg (A.T.); zhanina.petkova@orgchm.bas.bg (Z.P.); 2 Research and Development and Innovation Consortium, Sofia Tech Park, 1784 Sofia, Bulgaria; 3 Centre of Competence “Sustainable Utilization of Bio-Resources and Waste of Medicinal and Aromatic Plants for Innovative Bioactive Products” (BIORESOURCES BG), 1000 Sofia, Bulgaria; 4 Institute of Experimental Morphology, Pathology and Anthropology with Museum, Bulgarian Academy of Sciences, Acad. G. Bonchev Str., 25, 1113 Sofia, Bulgaria; taparsky@abv.bg; 5 Stephan Angeloff Institute of Microbiology, Bulgarian Academy of Sciences, Acad. G. Bonchev Str., 26, 1113 Sofia, Bulgaria; alexandraharalampieva@gmail.com; 6 National Diagnostic and Research Veterinary Medical Institute, bul. “Pencho Slaveikov” 15, 1606 Sofia, Bulgaria

**Keywords:** *Agrimonia eupatoria* L., HPLC–PDA–ESIMS, Orbitrap HRMS/MS, herpes simplex virus type 1, human coronavirus strain OC43, luminal A breast cancer cells (MCF-7), basal B-type breast cancer cells (MDA-MB-231), antiproliferative activity, phototoxicity

## Abstract

The limited spectrum of currently available antiviral drugs and the emergence of viral resistance highlight the need for novel antiviral agents with broad-spectrum activity and low toxicity. *Agrimonia eupatoria* L. (Rosaceae) is a medicinal plant rich in phenolic compounds with diverse biological activities. The present study investigated the phytochemical composition and antiviral and antiproliferative activities of methanolic (Ae-M) and hydromethanolic (Ae-WM) extracts of *A. eupatoria*. HPLC–HRMS/MS analysis revealed 28 phenolic constituents, predominantly flavonoid glycosides, flavan-3-ols, hydrolysable tannins and hydroxycinnamic acid derivatives. Semi-quantitative analysis revealed luteolin 7-*O*-glucoside, isoquercetin, hyperoside and agrimoniin as the major constituents. Both extracts exhibited low cytotoxicity and no detectable phototoxicity. Antiviral assays revealed inhibitory effects against herpes simplex virus type 1 (HSV-1) and human coronavirus OC43 (HCoV-OC43), with greater activity against HSV-1 (selectivity indices of 14.3–16.2 for both extracts). Additionally, both extracts exhibited pronounced virucidal activity against both viruses, reducing viral titers by Δlg = 2 to 3. They also completely inhibited HSV-1 adsorption to MDBK cells (with a reduction in Δlg = 5) and protected healthy cells from viral infection, with the strongest protective effect observed against HCoV-OC43, with a reduction in Δlg = 3.75. In addition, both extracts showed selective antiproliferative activity against MCF-7 and MDA-MB-231 breast cancer cells compared with non-tumorigenic MCF-12F cells. Overall, these findings identify *A. eupatoria* as a promising source of phenolic-rich extracts exhibiting in vitro antiviral and antiproliferative activities and support further investigation of their biological potential.

## 1. Introduction

Viral infections remain an important challenge to human health, while the effectiveness of antiviral therapy can be compromised by the limited availability of drugs for many viral diseases, treatment-associated toxicity, and the development of resistance to existing antiviral agents. The high mutation rates and adaptive capacity of viruses, particularly RNA viruses, facilitate the development of resistance and emphasize the continuing need for antiviral agents with novel mechanisms of action [1]. In this context, natural products provide access to a broad range of chemically diverse substances and have played a longstanding role in drug discovery. Plant-derived metabolites are of particular interest because they may interfere with different stages of viral infection, including virion attachment and entry, intracellular replication, and virus release, making medicinal plants attractive sources for antiviral investigation.

Among plant secondary metabolites, polyphenols have attracted particular interest due to their chemical diversity and wide spectrum of biological activities [1,2,3]. Flavonoids, phenolic acids, flavan-3-ols, proanthocyanidins, and hydrolysable tannins are widely distributed in medicinal plants and have been associated with antioxidant, anti-inflammatory, antimicrobial, and antiviral effects. Polyphenol-rich medicinal plants therefore represent promising sources of chemically diverse compounds for the identification of new antiviral agents.

*Agrimonia eupatoria* L. belongs to the Rosaceae family and is a perennial medicinal species, commonly referred to as agrimony, that has been used in traditional medicine for centuries. Preparations obtained from its aerial parts have been used for gastrointestinal disorders, including diarrhea and other digestive complaints, as well as for inflammation of the oral cavity and throat [4,5,6,7,8]. Topical preparations have also traditionally been applied to treat skin disorders, inflammation, wound healing, and their hemostatic effects [9,10,11,12]. In addition to these traditional applications, pharmacological studies have reported antidiabetic, antioxidant, neuroprotective, antibacterial, and antibiofilm activities of *A. eupatoria* extracts [13,14,15,16,17,18,19,20,21,22]. Nevertheless, the available pharmacological evidence is heterogeneous, and the relationships between extract composition, individual bioactive constituents, and specific biological effects remain incompletely understood [5]. Phytochemical investigations have identified phenolic acids, flavonoids, flavan-3-ols and procyanidins, and hydrolysable tannins in preparations obtained from the plant [5,23,24,25]. Frequently reported constituents include quercetin and kaempferol derivatives, catechin, procyanidins, and the ellagitannin agrimoniin. Importantly, the qualitative and quantitative phenolic composition of *A. eupatoria* preparations may vary according to the plant material and extraction procedure, emphasizing the importance of detailed chemical characterization when evaluating their biological properties [5]. Despite these reported biological activities, the antiviral potential of *A. eupatoria* remains relatively unexplored. *Kwon* et al. demonstrated that an aqueous extract prepared from the aerial parts of *A. eupatoria* inhibited hepatitis B virus surface antigen (HBsAg) release from HepG2.2.15 cells, providing evidence of antiviral activity against HBV [26]. However, information on the activity of *A. eupatoria* against other viruses and on the stages of viral infection targeted by its extracts remains limited.

The present study focused on the phytochemical characterization of sequential methanolic (Ae-M) and hydromethanolic (Ae-WM) extracts obtained from the aerial parts of A. eupatoria, together with an assessment of their antiviral and antiproliferative properties. Antiviral activity was examined using herpes simplex virus type 1 (HSV-1) and human coronavirus OC43 (HCoV-OC43) as experimental models, examining the effects of both extracts at different stages of infection. In addition, the antiproliferative activity and selectivity of the extracts were evaluated in breast cancer and non-tumorigenic breast cell lines. By integrating phytochemical characterization with biological evaluation, this study aimed to provide further insight into the bioactive potential of *A. eupatoria* and its phenolic-rich extracts.

## 2. Results and Discussion

### 2.1. Phytochemical Characterization of the Methanolic and Hydromethanolic Extracts of A. eupatoria

**Figure 1:**
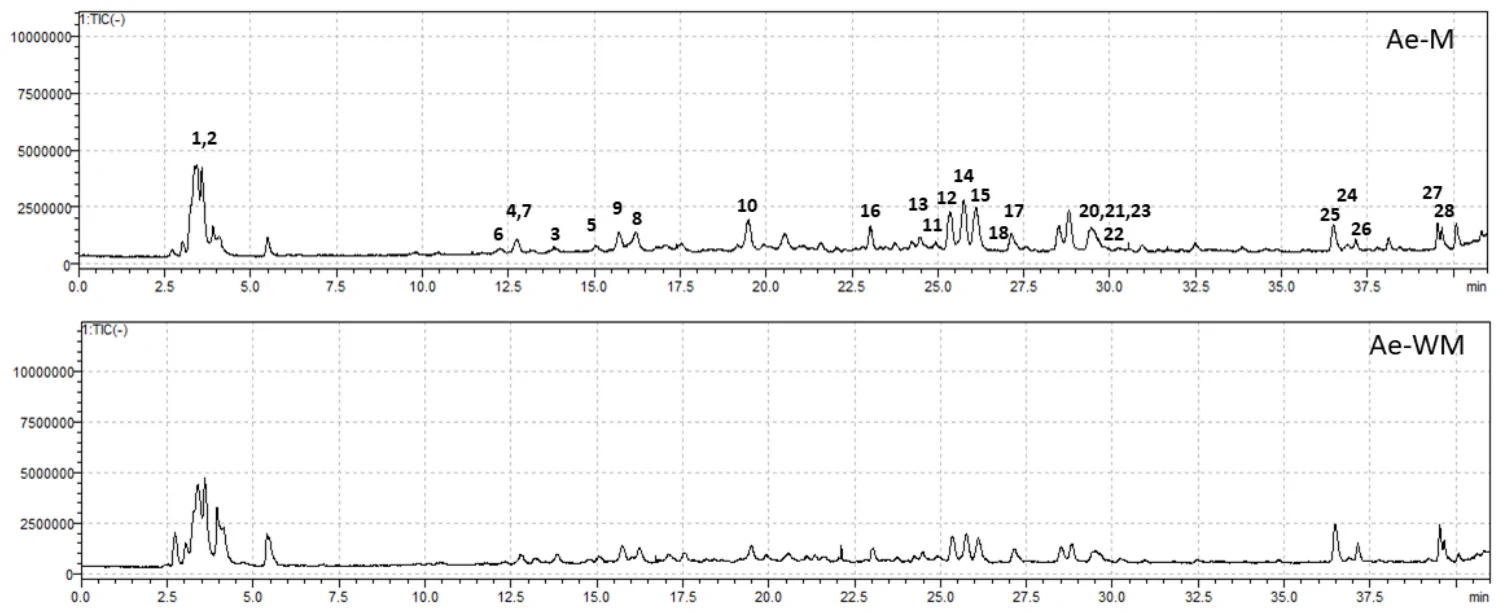
Total ion chromatograms (TIC) of the Ae-M and Ae-WM extracts of *A. eupatoria* acquired by HPLC–ESI/MS in negative ionization mode.

**Figure 2:**
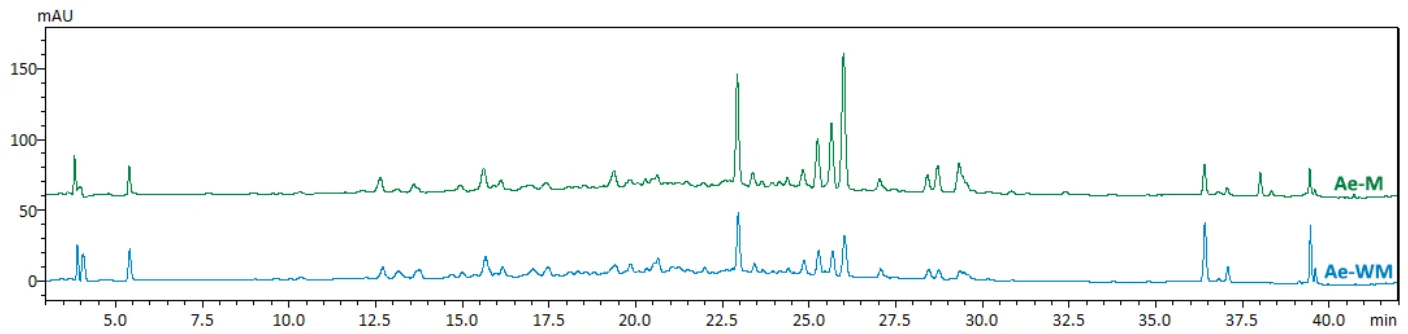
HPLC-PDA chromatograms of the Ae-M and Ae-WM extracts of *A. eupatoria* recorded at 280 nm.

**Figure 3:**
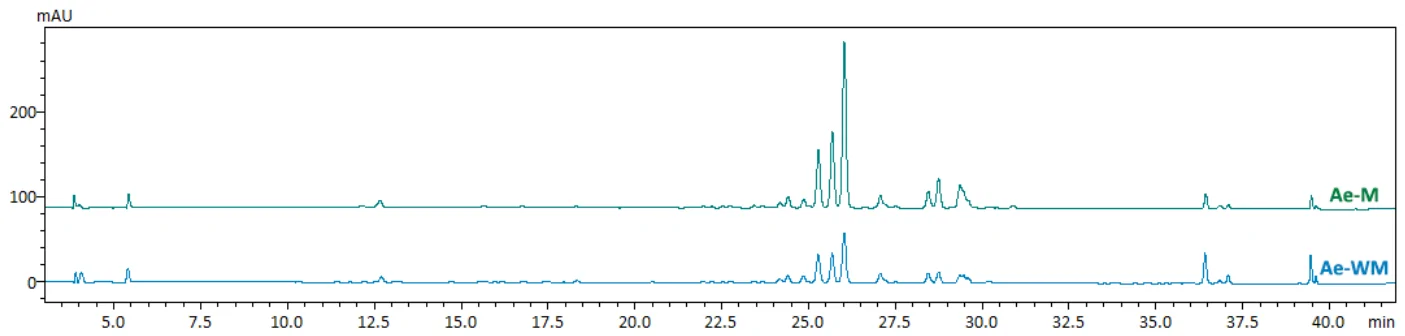
HPLC-PDA chromatograms of the Ae-M and Ae-WM extracts of *A. eupatoria* recorded at 350 nm.

Comprehensive phytochemical profiling of the methanolic (Ae-M) and hydromethanolic (Ae-WM) extracts of *A. eupatoria* was performed by HPLC-HRMS/MSand enabled the characterization of 28 phenolic constituents (Table 1). Compound identification was based on chromatographic retention times, accurate mass measurements, and MS/MS fragmentation patterns. Sixteen metabolites were confirmed using authentic reference standards, while the remaining compounds were tentatively identified through comparison with published analytical data. Representative total ion chromatograms (TIC) and HPLC-PDA chromatograms recorded at 280 and 350 nm are shown in Figure 1, Figure 2 and Figure 3.

The identified metabolites comprised four major phenolic classes, namely hydroxycinnamic acid derivatives, flavan-3-ols and procyanidins, hydrolysable tannins, and flavonoids. Hydroxycinnamic acid derivatives included neochlorogenic acid, chlorogenic acid isomer, 3-*O*-caffeoyl-5-*O*-malonylquinic acid and 3-*O*-*p*-coumaroylquinic acid. The flavan-3-ol fraction consisted of (+)-catechin, (−)-epicatechin and procyanidin oligomers, whereas the hydrolysable tannins were represented by pedunculagin, agrimoniin. Ellagic acid was also identified in both extracts. Agrimoniin was the predominant ellagitannin detected in both extracts. Flavonoids represented the largest and most structurally diverse metabolite group, comprising glycosides of quercetin, kaempferol, luteolin and apigenin, together with several acylated derivatives, including *trans*- and *cis*-tiliroside and acetyl-*p*-coumaroyl kaempferol glucosides.

Although Ae-M and Ae-WM showed highly similar qualitative phytochemical profiles, quantitative HPLC-PDA-ESIMS analysis revealed notable differences in the abundance of individual constituents (Table 1, Figure 2 and Figure 3). Overall, Ae-M contained higher concentrations of several phenolic compounds than Ae-WM, with flavonoid glycosides representing the predominant group in both extracts. Luteolin 7-*O*-glucoside was the most abundant quantified constituent in Ae-M (30.22 mg HypE/g DE), followed by isoquercetin (13.16 mg HypE/g DE), hyperoside(10.36 mg HypE/g DE), agrimoniin (7.63 mg EA/g DE), and quercitrin (4.97 mg HypE/g DE). Higher concentrations of catechin, epicatechin, procyanidins, and several kaempferol glycosides were also observed in Ae-M. In contrast, Ae-WM contained comparatively higher amounts of some acylated flavonoid glycosides, particularly *cis-* and *trans*-tiliroside and kaempferol derivatives **27** and **28**.

The quantitative differences between Ae-M and Ae-WM are likely related to the sequential extraction procedure. Following the initial removal of non-polar constituents with dichloromethane, extraction with methanol recovered higher amounts of several flavonoid glycosides, flavan-3-ols, and hydrolysable tannins than the subsequent methanol-water extraction. Despite these quantitative differences, the principal phenolic classes were represented in both extracts, indicating that the sequential extraction procedure affected primarily the relative abundance of individual constituents rather than the overall qualitative phytochemical profile.

The phytochemical profiles established in the present study are generally consistent with previous reports describing *A. eupatoria* as a rich source of flavonoids, flavan-3-ols, phenolic acids, and hydrolysable tannins, including quercetin and luteolin glycosides and the characteristic ellagitannin agrimoniin [25,27,28,29,30]. The predominance of luteolin 7-*O*-glucoside, isoquercetin, hyperoside, and agrimoniin in the investigated extracts further confirms the abundance of phenolic constituents in this species. Importantly, the observed quantitative differences between Ae-M and Ae-WM provide a basis for examining potential associations between extract composition and the biological activities evaluated in the following sections.

### 2.2. Cytotoxicity and Phototoxicity of A. eupatoria Extracts

The cytotoxicity and phototoxicity of the *A. eupatoria* extracts were initially evaluated in BALB 3T3 cells, with phototoxicity assessed using the 3T3 neutral red uptake (NRU) assay. In addition, the maximum non-toxic concentrations of both extracts were determined in MDBK and Vero E6 cells, which were subsequently used in the antiviral assays. Toxicity was calculated using the following formula: Toxicity (%) = (1 − (OD_540_ (sample)/OD_540_ (negative control))) × 100.

**Figure 4:**
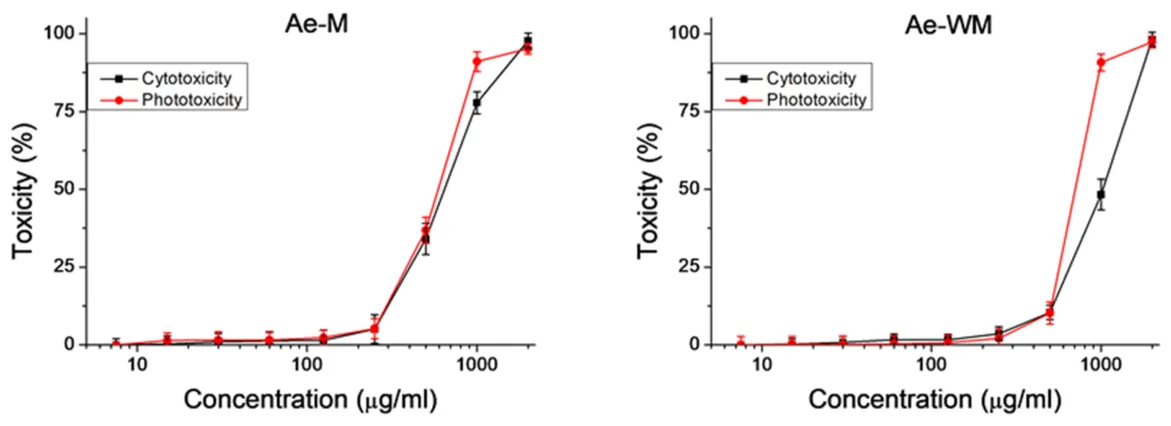
Cytotoxicity and phototoxicity of *A. eupatoria* extracts: Ae-M and Ae-WM, determined in BALB 3T3 clone A31 cell line. Toxicity is presented as a percentage relative to the negative control. Data were obtained from three independent experiments, *n* = 6.

The results are presented in Figure 4 as dose–response curves, while Table 2 summarizes the mean TC_50_ values (50% toxic concentration). Both extracts exhibited low toxicity, with average values of TC_50_ = 642.23 ± 31.16 µg/mL for Ae-M and 1020.53 ± 70.93 µg/mL for AE-WM. The phototoxicity of the extracts was evaluated using the Photo-Irritation Factor (PIF), where PIF = (TC_50_ − Irr)/(TC_50_ + Irr). The PIF values for both extracts were lower than 2, indicating that neither extract was phototoxic under the experimental conditions.

**Figure 5:**
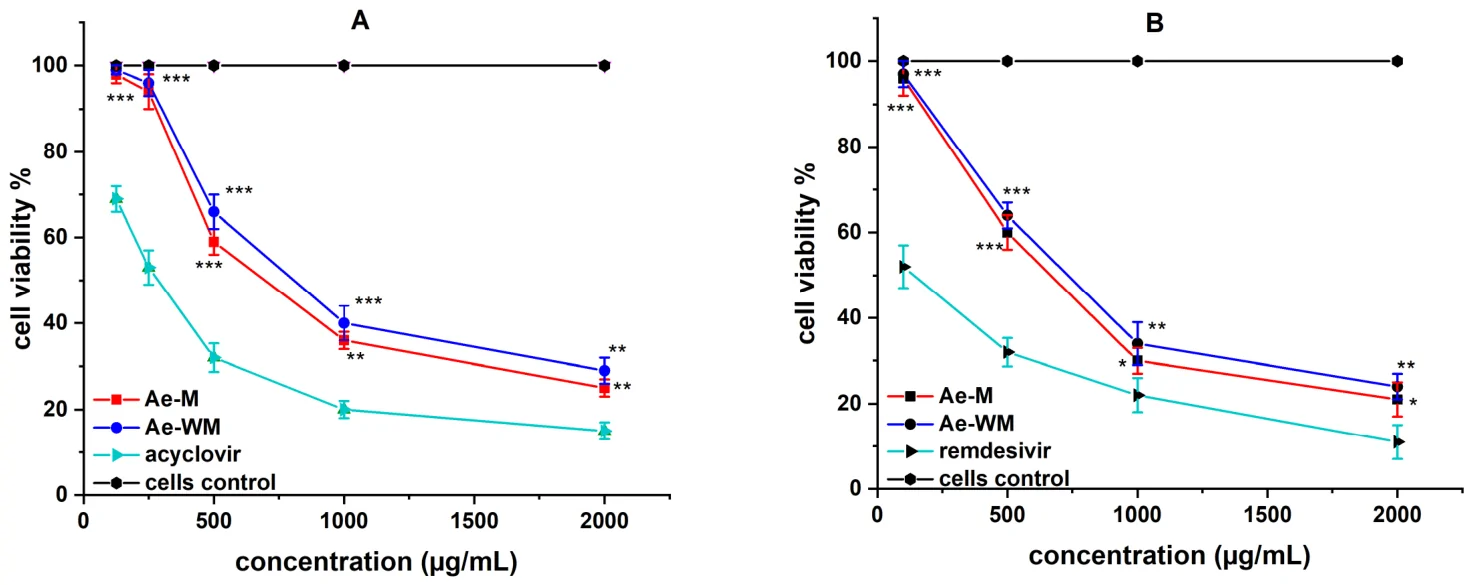
Cytotoxic effect of *A. eupatoria* extracts: (**A**) against MDBK cell line; (**B**) against Vero E6 cell line. * *p* < 0.05, when comparing the value of each sample with the corresponding reference substance; ** *p* < 0.01, when comparing the value of each sample with the corresponding reference substance; *** *p* < 0.001, when comparing the value of each sample with the corresponding reference substance; one-way ANOVA, followed by Bonferroni’s post hoc test; *n* = 3.

Comparison of the cytotoxic effects in MDBK and Vero E6 cells revealed slightly greater cytotoxicity in the latter cell line. This difference may be associated with the longer incubation period used for Vero E6 cells (120 h) than for MDBK cells (48 h). The incubation period was selected according to the time required to achieve an optimal cytopathic effect in the antiviral assays. Of the two tested extracts, Ae-WM exhibited slightly lower cytotoxicity toward both cell lines with CC_50_ = 796.3 and 737.9 for MDBK and Vero E6 cell lines, respectively. Both extracts exhibited significantly lower cytotoxicity than the corresponding reference compounds (Table 3 and Figure 5).

### 2.3. Antiviral Activity

**Figure 6:**
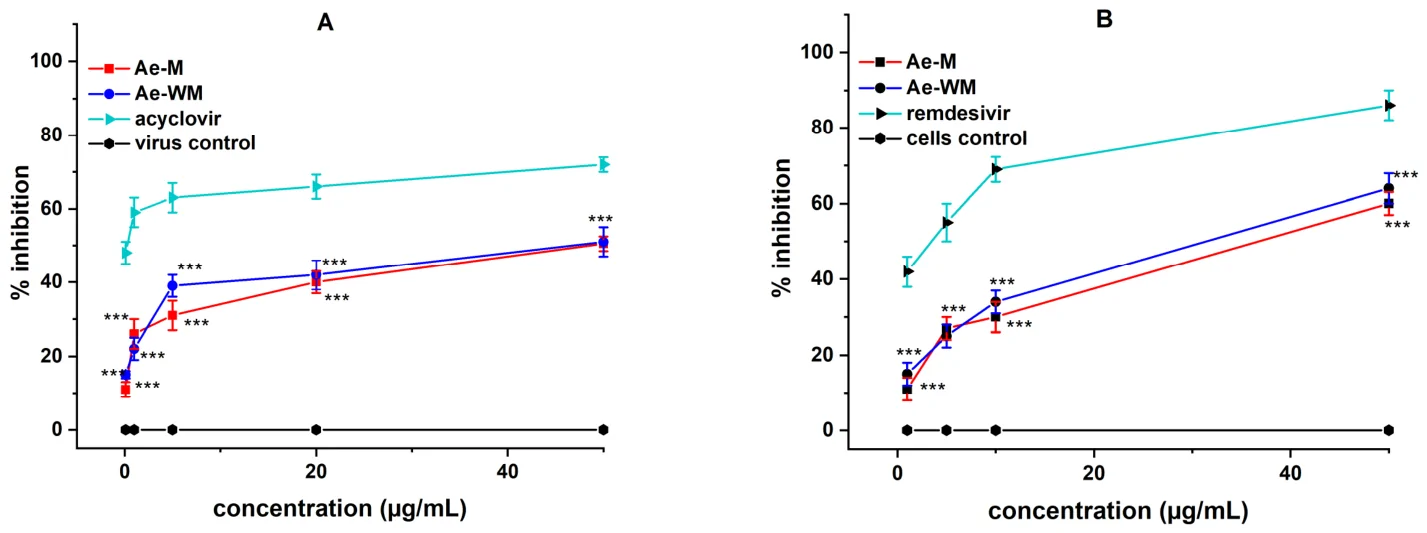
Effect on viral replication of *A. eupatoria* extracts—(**A**) against HSV-1; (**B**) against HCoV-OC43. *** *p* < 0.001—when comparing the value of each sample with the corresponding reference substance; one-way ANOVA, followed by Bonferroni’s post hoc test; *n* = 3.

The effects of the two *A. eupatoria* extracts on the replication of the two tested viruses were investigated. Both extracts demonstrated inhibitory effects against HSV-1 replication. The effect was slightly more pronounced for Ae-WM with SI = 16.2 compared to Ae-M with SI = 14.3. Regarding HCoV-OC43 replication, Ae-M demonstrated slightly stronger antiviral activity, with an SI of 7.4, compared to Ae-WM (SI = 5.1) (Table 3 and Figure 6).

**Figure 7:**
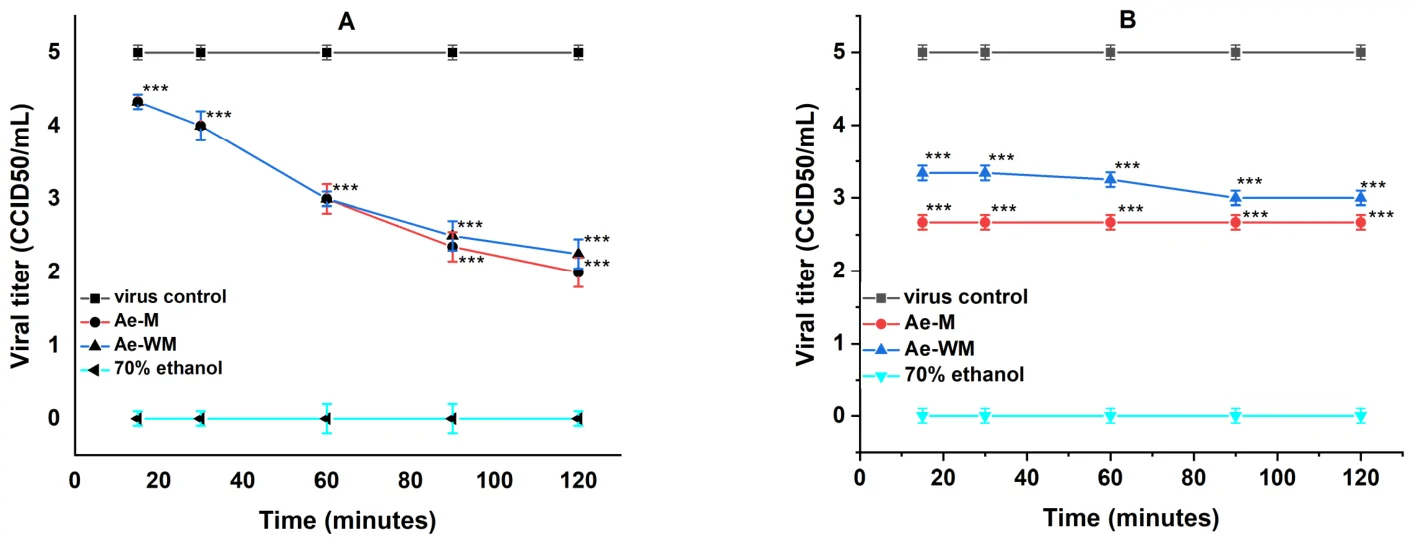
Effect of *A. eupatoria* extracts on extracellular virions: (**A**) against HSV-1 virions; (**B**) against HCoV-OC43 virions. *** *p* < 0.001, when comparing the value of each sample with the values obtained for the reference substance 70% ethanol; one-way ANOVA, followed by Bonferroni’s post hoc test; *n* = 4.

Following the evaluation of antiviral activity against viral replication, the virucidal effects of both extracts were investigated. In the case of HSV-1 virions, the effect was distinct and time-dependent—the effect improved with increasing exposure time. The activity was determined at different time intervals. At the first two-time intervals (15 and 30 min), neither extract showed a significant inhibitory effect. At 60 min of exposure, both extracts caused distinct inhibition of the virions, which led to a decrease in viral titers by Δlg = 2.0. This was followed by an increase in activity, which led to a decrease in viral titers at 120 min by Δlg = 3.0 for Ae-M and Δlg = 2.75 for Ae-WM (Table 4 and Figure 7A).

In contrast to HSV-1, the virucidal activity against HCoV-OC43 showed no pronounced dependence on exposure time. Ae-M exhibited a stronger effert, reducing viral titers with Δlg = 2.33 at all studied time intervals. The activity of Ae-WM was slightly lower but also significant, with an initial Δlg = 1.75, which reached Δlg = 2.0 at the final time intervals (Table 4 and Figure 7B).

**Figure 8:**
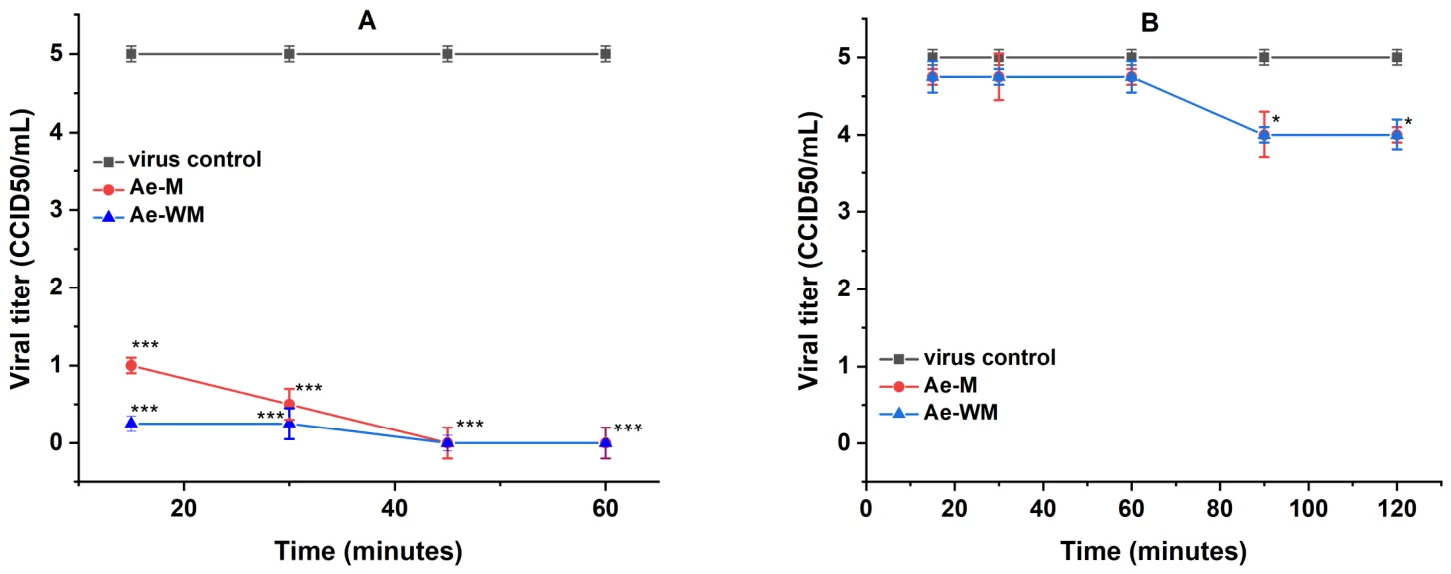
Effects of *A. eupatoria* extracts on the viral adsorption stage: (**A**) of HSV-1 to MDBK cells; (**B**) of HCoV-OC43 to Vero E6 cells. * *p* < 0.05, *** *p* < 0.001, when comparing the value of each sample with the viral control; one-way ANOVA, followed by Bonferroni’s post hoc test; *n* = 4.

Following the evaluation of the effect on extracellular virions, the influence of *A. eupatoria* extracts on viral adsorption to susceptible cells was investigated. During the adsorption of HSV-1 to MDBK cells, the process was remarkably inhibited already at the first monitored time interval (15 min) with a decrease in viral titers by Δlg = 4.75 in Ae-WM and by Δlg = 4.0 in Ae-M. After 45 min, the process was blocked 100% (Δlg = 5.0) and no cytopathic effect was observed in the cell monolayer. In contrast, neither extract significantly inhibited HCoV-OC43 adsorption to Vero E6 cells during the investigated period of up to 120 min (Table 5 and Figure 8).

**Figure 9:**
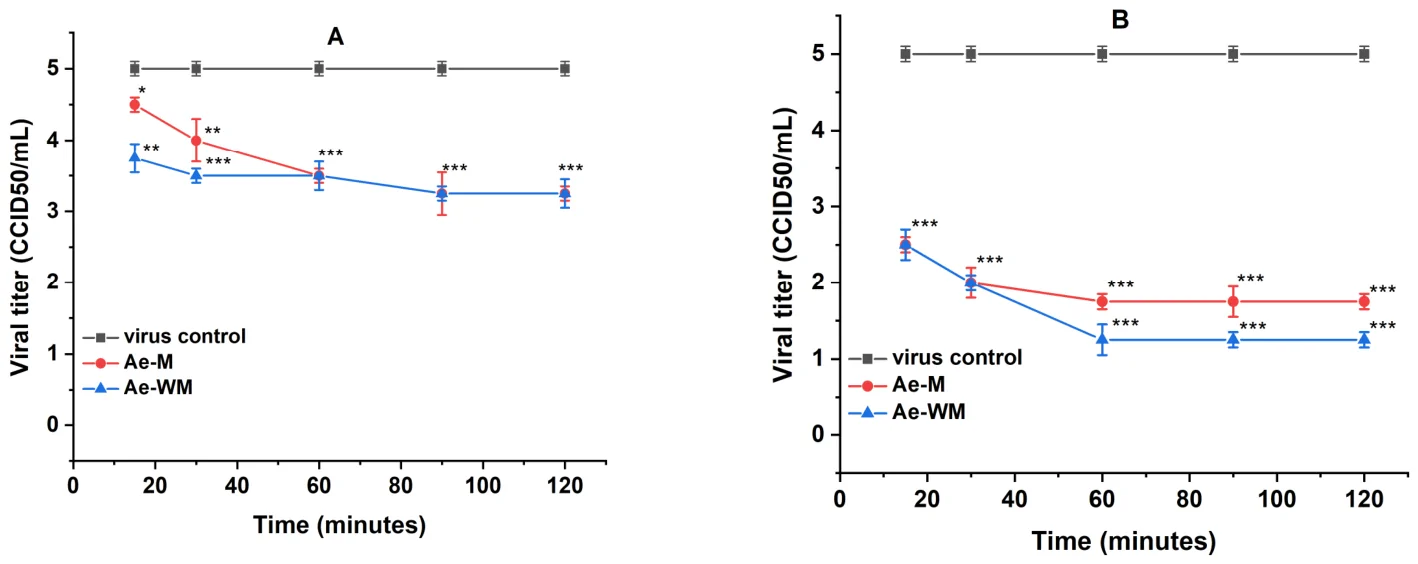
Protective effect of *A. eupatoria* extracts on healthy cells before viral infection: (**A**) of MDBK cells before HSV-1 infection; (**B**) of Vero E6 cells before HCoV-OC43 infection. * *p* < 0.05, ** *p* < 0.01, *** *p* < 0.001, when comparing the value of each sample with the viral control; one-way ANOVA, followed by Bonferroni’s post hoc test; *n* = 4.

After investigating the stages of HSV-1 and HCoV-OC43 replication affected by *A. eupatoria* extracts, their protective effects on uninfected MDBK and Vero E6 cells against subsequent viral infection were evaluated. In both cell lines, the protective effect increased with longer pretreatment periods. In MDBK cells, a significant protective effect was observed at 90 and 120 min of pretreatment of the cells (Δlg = 1.75). A more pronounced protective effect was observed in Vero E6 cells, where both extracts reduced HCoV-OC43 titers by Δlg = 2.5 after only 15 min of pretreatment. The effect was enhanced with longer pretreatment and at 120 min of exposure, a decrease in virus titers was reached to Δlg = 3.75 for Ae-WM and Δlg = 3.25 for Ae-M (Table 6 and Figure 9).

Several phenolic constituents identified in the investigated *A. eupatoria* extracts have previously been associated with antiviral activities in studies involving other viruses and experimental models. Among these, two compounds (+)-catechin and (−)-epicatechin, which belong to the group of catechins, have attracted attention for their potential antiviral properties. Previous studies have demonstrated antiviral activity of various catechins against diverse viral pathogens, including dengue, Mayaro virus, influenza, SARS-CoV-2, Singapore Grouper Iridovirus, HIV-1 and hepatitis. These effects have been observed at various stages of viral infection, including viral entry, nucleic acid replication, and viral protein synthesis. Inhibition of SARS-CoV-2 attachment to host–cell ACE2 receptors through its spike protein has been demonstrated. A similar effect has been observed for the interaction between HIV-1 gp120, which may be substantially inhibited. In HBV, inhibition of the production of the viral HBeAg was observed, as well as an influence on the replication of the viral DNA [31,32,33,34,35,36,37]. Hyperoside, another constituent identified in the investigated extracts, has also been associated with antiviral activity in previous studies. Reported mechanisms of action include blocking the attachment and entry of the virus into host cells. Hyperoside has been shown to interact with the hemagglutinin protein of influenza A virus (IAV), thereby inhibiting viral attachment to host cells. This compound has also been reported to inhibit the adsorption and internalization of EHV-8. Furthermore, it suppresses the replication of hepatitis B virus (HBV) DNA and inhibits the secretion of HBsAg and HBeAg in liver cells. Against hepatitis C (HCV), it demonstrates activity by inhibiting the NS3 protease [38,39,40]. Isoquercetin is known from previous studies to show antiherpes and anticoronavirus potential, as well as to affect viral adsorption in Influenza A virus by modulating hemagglutinin and neuraminidase [41,42,43]. Luteolin-7-O-glucoside has been reported to exhibit anti-coronavirus activity through a dual mechanism. On the one hand, it directly affects viral proteins and reduces the ability of the virus to attach and enter the cell. On the other hand, it modulates the mechanisms of the host cells to block the penetration of the virus [43,44,45]. Agrimoniin, a characteristic ellagitannin of A. eupatoria, has also been reported to exhibit antiviral activity against several viruses, including hepatitis B virus (HBV), influenza A virus and SARS-CoV-2. The effect was observed both at the stage of viral replication and at the viral surface proteins responsible for the attachment of the virus to the cell surface, thereby blocking viral entry into the cell [46,47].

The antiviral properties previously reported for several of the identified phytochemical constituents provide useful context for the present findings. However, the biological activities observed in this study were evaluated using crude extracts, and the contribution of individual compounds cannot be determined from the present results. The inhibition of viral replication, together with the observed virucidal and anti-adsorption effects, suggests that the extracts may affect different stages of viral infection. In particular, the pronounced inhibition of HSV-1 adsorption indicates an effect on the early stages of infection, while the protective activity observed following pretreatment of uninfected cells, especially against HCoV-OC43, suggests that the extracts may also influence cellular susceptibility to infection. Nevertheless, these observations do not establish the molecular mechanisms underlying the antiviral effects.

### 2.4. Determination of Anti-Proliferative Activity

**Figure 10:**
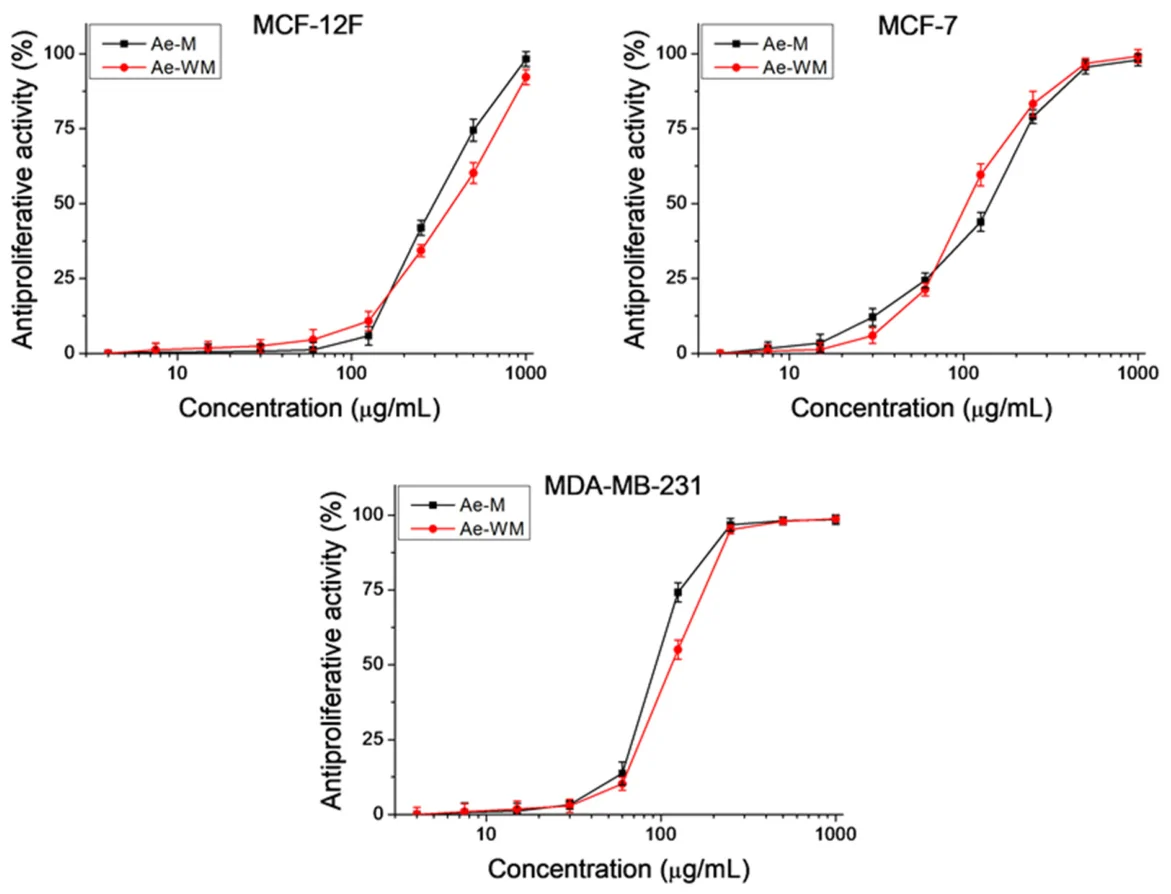
Antiproliferative activity of the extracts Ae-M and Ae-WM, in the non-tumorigenic cell line MCF-12F (model of healthy tissue) and the tumour cell lines MCF-7 and MDA-MB-231 (in vitro model of breast cancer). Antiproliferative activity is presented as a percentage relative to the negative control. Data were obtained from three independent experiments; *n* = 6.

To further characterize the biological activity of the studied extracts, their effects were evaluated not only in normal cells but also in terms of their antiproliferative effect against several tumor cell lines. The antiproliferative activity of the Ae-M and Ae-WM extracts was investigated using the MTT assay. Antiproliferative activity is presented as a percentage of the negative control and is calculated using the following formula: Antiproliferative activity (%) = (1 − (OD_540_ (sample)/OD_540_ (negative control))) × 100. Based on the results obtained, dose–response curves were generated (Figure 10). The calculated selectivity index (SI) and mean IC_50_ values are presented in Table 7. The tested extracts exhibited higher antiproliferative activity in tumor cells compared to non-tumorigenic MCF-12F cells. The highest antiproliferative activity was observed for extract Ae-M against triple-negative breast cancer (MDA-MB-231) with an average value of IC_50_ = 93.3 ± 3.79 µg/mL. However, extract Ae-WM exhibited the highest selectivity toward luminal type A breast cancer cells (MCF-7) with SI = 3.66.

Overall, these findings highlight the antiviral potential of *A. eupatoria* extracts, which exerted effects at different stages of viral infection. Their antiviral activity, together with low cytotoxicity and the absence of phototoxicity under the experimental conditions, supports further investigation of *A. eupatoria* as a source of bioactive constituents.

## 3. Materials and Methods

### 3.1. Plant Material

The plant material, consisting of the aerial parts of *A. eupatoria*, was harvested in July 2023 from Golo Burdo Mountain, Bulgaria (42°30′38″ N, 23°04′45″ E). Following collection, the material was air-dried, ground, and stored in the dark until extraction. Botanical identification was carried out by Prof. Dr. Ina Aneva of the Institute of Biodiversity and Ecosystem Research, Bulgarian Academy of Sciences (IBER-BAS), Sofia, Bulgaria. A voucher specimen (SOM 1438) was deposited in the herbarium of IBER-BAS.

### 3.2. Extraction Procedure

Dried aerial parts of *A. eupatoria* (10 g) were subjected to sequential ultrasound-assisted extraction with dichloromethane, methanol and methanol-water (1:1, *v*/*v*). Each extraction was performed with 400 mL of solvent in an ultrasonic bath (Bandelin Sonorex Digiplus DL 255 H, Berlin, Germany) at room temperature in three consecutive 30 min extraction cycles. Following each extraction step, the solvent was removed under reduced pressure using a rotary evaporator (Büchi Labor-Technik AG, Flawil, Switzerland) to obtain the corresponding dry extract. Three extracts were obtained: a dichloromethane extract (Ae-D, 0.29 g, 2.9% DW), a methanolic extract (Ae-M, 1.63 g, 16.3% DW), and a hydromethanolic extract (Ae-WM, 1.53 g, 15.3% DW). Extraction yields were expressed as the percentage of the dry extract mass relative to the initial dry weight (DW) of the plant material.

The resulting extracts were kept at 4 °C until further analysis. The initial dichloromethane extraction was performed to remove lipophilic constituents prior to extraction of the more polar metabolites. The present study focused on the methanolic (Ae-M) and hydromethanolic (Ae-WM) extracts.

### 3.3. Qualitative Phytochemical Profiling by LC–HRMS/MS

The qualitative chemical composition of the extracts was performed using a Q Exactive Plus^®^ hybrid quadrupole-Orbitrap^®^ high-resolution mass spectrometer equipped with heated electrospray ionization (HESI) source and interfaced with a Vanquish HPLC system (Thermo Fisher Scientific, Bremen, Germany). An Accucore™ C18 analytical column (150 × 2.1 mm, 2.6 μm, Thermo Fisher Scientific™, Germany) was used for chromatographic separation. The mobile phase comprises two components: aqueous 0.1% (*v/v*) formic acid containing 5% (*v/v*) acetonitrile (A) and acetonitrile containing 0.1% (*v/v*) formic acid and 5% (*v/v*) water (B). Gradient elution started with 5% B, maintained for the first 2 min, followed by a linear increase to 30% B over the next 23 min. The proportion of B was subsequently raised to 95% between 25 and 30 min and held constant until 34 min. The initial composition (5% B) was restored between 34 and 35 min, followed by a 5 min re-equilibration period. The chromatographic separation was performed over 40 min at a flow rate of 0.3 mL/min, using an injection volume of 3 μL. The HESI source settings included a spray voltage of 2.90 kV and a capillary temperature of 320 °C. Sheath, auxiliary, and sweep gas flows were set to 30, 6, and 0 arbitrary units, respectively, while the S-lens RF level was adjusted to 50 V. Nitrogen served as the nebulizing gas and as the collision gas in the HCD cell. Mass spectra were recorded in full-scan negative ionization mode within an *m/z* range of 120–1200, at a resolution of 70,000. The AGC target was set to 1 × 10^6^, with a maximum ion injection time of 80 ms. For data-dependent acquisition (Top5), the five highest-intensity precursor ions detected in each survey scan were subjected to MS/MS fragmentation. The ddMS^2^ acquisition settings included a resolving power of 17,500, an AGC target of 1 × 10^5^, a maximum injection time of 50 ms, a precursor isolation width of 2.0 *m/z*, and stepped normalized collision energies (NCE) of 20, 40, and 70. Data acquisition was controlled through Xcalibur 4.2 SP1, whereas subsequent data processing was carried out using FreeStyle 1.5 (Thermo Fisher Scientific). The *A. eupatoria* dry extracts, Ae-M and Ae-WM, were initially dissolved in methanol and a 50% *(v/v*) water-methanol mixture, respectively, to a concentration of 5000 mg/L, and subsequently diluted with a 50% (*v/v*) water-methanol mixture to a final concentration of 100 mg/L. Prior to analysis, both extracts were passed through hydrophilic polytetrafluoroethylene (PTFE) syringe membrane filters with a diameter of 13 mm and a pore size of 0.22 μm (VWR International, Radnor, PA, USA).

### 3.4. Semi-Quantitative HPLC-PDA-ESI/MS Analysis

The HPLC-PDA-ESI/MS measurements were conducted on a Shimadzu LC-2040C 3D Nexera platform equipped with an LCMS-2020 single-quadrupole mass detector (Shimadzu, Tokyo, Japan). The compounds were separated on a Restek Force C18 column (150 × 4.6 mm, 3 µm; Restek, Bellefonte, PA, USA), maintained at 40 °C throughout the analysis. The elution system comprised aqueous formic acid (0.1%, *v/v*) as solvent A and acetonitrile as solvent B. Gradient elution was performed by progressively increasing the proportion of solvent B from 5% to 30% over the first 30 min, followed by an increase to 45% between 30 and 35 min and subsequently to 95% between 35 and 37 min. This composition was maintained for 6 min (37–43 min), after which the initial conditions (5% B) were restored within 1 min. The column was then re-equilibrated at 5% B for 7 min, resulting in a total analytical run time of 51 min. UV–Vis spectra were recorded between 190 and 800 nm, using a flow rate of 0.4 mL/min and an injection volume of 5 µL. Mass spectrometric detection was conducted in negative electrospray ionization (ESI−) mode, applying a spray voltage of −3.50 kV. Mass spectra were collected over an *m/z* range of 100–1000. The interface, desolvation line, and heat block temperatures were set to 350, 250, and 200 °C, respectively, while the nebulizing and drying gas flow rates were adjusted to 1.5 and 15 L/min, respectively. Chromatographic and mass spectrometric data were processed using LabSolutions software (version 5.128 SP1; Shimadzu Corporation, Kyoto, Japan, 2008–2024). Individual compounds were identified based on their retention times, UV–Vis spectra, and mass spectrometric data, supported by comparison with reference standards and previously published literature. Semi-quantitative estimation of phenolic compounds in the two extracts was performed using external calibration with ellagic acid and hyperoside as representative reference standards. The resulting concentrations were expressed as equivalents of the corresponding reference standards and should be interpreted as approximate values rather than absolute concentrations of individual compounds. Calibration curves were constructed using standard solutions of ellagic acid at concentrations of 10, 50, and 100 mg/L and hyperoside at 1, 10, 25, 50, and 100 mg/L. The corresponding linear regression equations were y = 34,378x − 16,451 (R^2^ = 0.999) for ellagic acid and y = 23,156x − 751.04 (R^2^ = 0.999) for hyperoside. Prior to chromatographic analysis, Ae-WM and Ae-M were prepared at a concentration of 2000 mg/L using methanol/water and methanol as solvents, respectively. The semi-quantitative determination of quercetin and kaempferol glycosides was carried out at 350 nm using the hyperoside calibration curve, with the results expressed as milligrams of hyperoside equivalents per gram of extract (mg HypE/gE). Procyanidins, hydroxycinnamic acid derivatives, catechins, ellagitannins, and ellagic acid were quantified at 280 nm based on the calibration curve of ellagic acid, and their concentrations were reported as milligrams of ellagic acid equivalents per gram of extract (mg EA/gE).

### 3.5. Reagents and Standards

Authentic standards, including quinic acid (≥98.0%, CAS: 77-95-2), neochlorogenic acid (≥95.0%, CAS: 906-33-2), (+)-catechin (≥98.0%, CAS: 154-23-4), (-)-epicatechin (≥98.0%, CAS: 490-46-0), ellagic acid (≥95.0%, CAS: 476-66-4), quercetin 3-*O*-galactoside (hyperoside, ≥95.0%, CAS: 482-36-0), rutin (≥95.0%, CAS: 153-18-4), quercetin 3-*O*-glucoside (isoquercetin, ≥95.0%, CAS: 482-35-9), luteolin 7-*O*-glucoside (≥95.0%, CAS: 5373-11-5), kaempferol 3-*O*-rutinoside (≥95.0%, CAS: 17650-84-9), kaempferol 3-*O*-glucoside (≥95.0%, CAS: 480-10-4), quercetin 3-*O*-rhamnoside (quercitrin, ≥95.0%, CAS: 522-12-3), apigenin 7-*O*-glucuronide (≥95.0%, CAS: 29741-09-1), apigenin 7-*O*-glucoside (≥95.0%, CAS: 578-74-5), luteolin (≥95.0%, CAS: 491-70-3), *trans*-tiliroside (kaempferol-3-*O*-(6″-*O*-*p*-coumaroyl)-glucoside, ≥95.0%, CAS: 20316-62-5) were purchased from PhytoLab GmbH & Co. KG (Vestenbergsgreuth, Germany).

LC-MS-grade acetonitrile and methanol (Chromasolv^®^) were obtained from Honeywell Riedel-de Haën (Seelze, Germany), whereas formic acid was sourced from Sigma-Aldrich (Buchs, Switzerland). Ultrapure water was generated in-house using a Smart2Pure 12 UV/UF purification system (Thermo Electron LED GmbH, Langenselbold, Germany).

### 3.6. Cells and Viruses

#### 3.6.1. Host Cell Lines

From the collection of the Institute of Microbiology, Bulgarian Academy of Sciences, Sofia, Bulgaria—Vero E6 cell line and Madin-Darby bovine kidney (MDBK) cells.

Purchased from the American Type Culture Collection (ATCC, Manassas, VA, USA)—“Luminal A” type breast cancer cells (line MCF-7), “Basal B” type breast cancer cells (line MDA-MB-231), mouse embryonic fibroblasts (line BALB 3T3, clone A31) and human mammary epithelial cells (line MCF-12F).

#### 3.6.2. Viruses

Human coronavirus strain OC43 (HCoV-OC43) (ATCC: VR-1558) (Manassas, VA, USA) was cultured in Vero E6 cells. The virus was titrated according to the Reed and Muench formula [48]. The infectious titer of the viral stock was 10^6.5^ CCID50/mL.

Herpes simplex virus type 1 (HSV-1), was cultured in MDBK cells (provided by the collection of the Institute of Microbiology at the Bulgarian Academy of Sciences). The infectious viral titer was 10^8.5^ CCID_50_/mL.

#### 3.6.3. Reference Compound

To compare antiviral activity against the viral replicative cycle of the viruses under study, some of the most effective agents—proven in the treatment of the respective viruses—were used.

Double-distilled water was used for the initial dissolution of Remdesivir (GS-5734, RDV, REM, Veklury^®^) (Gilead Science Ireland UC, Dublin, Ireland). Subsequent dilutions were prepared using the culture medium (DMEM) in which the cells were cultured. Remdesivir was used to compare the observed antiviral activity against HCoV-OC43.

The initial concentration of acyclovir (ACV, 9-(2-hydroxyethoxymethyl)-guanine) was 3 mM in DMSO. It was kindly provided by the Deutsches Krebsforschungszentrum (German Cancer Research Center), Heidelberg. Serial dilutions were prepared in DMEM medium. Acyclovir was used as a reference substance for comparing antiviral activity against HSV-1.

The reference substance chlorpromazine (Sigma-Aldrich, Schnelldorf, Germany) was used as a positive control in the photosafety test. Chlorpromazine is used to treat some mental illnesses and is highly phototoxic. Cisplatin (Sigma-Aldrich, Schnelldorf, Germany) was used as a reference substance in the antiproliferative activity test. Cisplatin is widely used in oncology as a drug that inhibits the proliferation of tumor cells.

### 3.7. Cytotoxicity Assay

Cells from the various cell lines were seeded into 96-well plates (Costar^®^, Corning Inc., Kennebunk, ME, USA). The cells were treated with 0.1 mL/well of various concentrations of *A. eupatoria* extracts. They were incubated at 37 °C and 5% CO_2_, after which the extent of Neutral Red (NR) dye uptake was determined. The concentration at which the applied extract reduced cell viability by 50% (CC_50_) was determined [49]. Each sample was analyzed in three independent replicates, and the arithmetic mean value was determined (n = 3). Within each replicate, every sample was tested in four-well replicates, from which the arithmetic mean value was subsequently calculated based on the obtained data. The maximally tolerated concentration (MTC) of the tested samples, at which the morphology of the cell monolayer was not affected, was also determined.

### 3.8. Photosafety Test

BALB 3T3 cells were treated with different concentrations of extracts and irradiated with a solar simulator (Helios-iO, SERIC Ltd., Tokyo, Japan). Phototoxicity assay [50] was used and viability was determined by Neutral Red Uptake (NRU) assay [49]. Phototoxicity was calculated according to the following formula:

Toxicity (%) = (1 − (OD_540_ (sample)/OD_540_ (negative control))) × 100

The Photo-Irritation Factor (PIF) was also calculated.

PIF = TC_50_ −Irr/TC_50_ +Irr*, * Irr (Irradiation)

### 3.9. Determination of Infectious Viral Titers

Cells from Vero E6 or MDBK cell lines were infected with 0.1 mL of a viral suspension, subjected to ten-fold serial dilutions, in 96-well plates. Following the viral adsorption period, the unadsorbed virus was removed. After incubation, cytopathic effect (CPE) was determined microscopically and confirmed by neutral red uptake assay [49].

### 3.10. Antiviral Activity Assay

A cytopathic effect (CPE) inhibition assay [51] was used to determine the antiviral activity of the extracts. A cell monolayer was covered with a viral suspension and incubated for the duration of the viral infection. Unadsorbed virus was removed, and the extracts were added at various concentrations. Following incubation, the cytopathic effect was determined via neutral red uptake. The inhibitory effect was determined using the following formula:

%CPE = [OD _test sample_ − OD _virus control_]/[OD _toxicity control_ − OD _virus control_] × 100,

where the OD_test sample_ is the mean of wells containing virus and treated with the extract; the OD_virus control_ is virus with no added compound; the OD _toxicity control_ is the optical density in the wells that are treated with the corresponding concentration of the extract but are not infected with the virus.

Each sample was analyzed in three independent replicates, and the arithmetic mean value was determined (n = 3). Within each replicate, every sample was tested in four-well replicates, from which the arithmetic mean value was subsequently calculated based on the obtained data.

### 3.11. Effect on Viral Adsorption

The cells were simultaneously inoculated with 10^5^ CCID_50_ of the respective virus and treated with the test extracts at their MTC. This is followed by an incubation period to allow for viral adsorption. At various time intervals (Table 5), the virus and the extract are removed. A subsequent incubation period follows, allowing for the development of a cytopathic effect. The viral titer was determined, and Δlg values were calculated. Each sample was analyzed in four independent replicates, and the arithmetic mean value was determined (n = 4). In each replicate, every sample was represented by four individual wells in the cell culture plate.

### 3.12. Virucidal Assay

The extract (at its MTC) and the virus are mixed in equal quantities. The samples are incubated for various time intervals (Table 4), after which the viral titers are determined. The Δlog values are calculated. Each sample was analyzed in four independent replicates, and the arithmetic mean value was determined (n = 4). In each replicate, every sample was represented by four individual wells in the cell culture plate.

### 3.13. Pre-Treatment of Healthy Cells

The cell monolayer was treated for different time intervals with the extract (in its MTC). It was followed by infection with the corresponding virus strain, which was incubated for the time of viral adsorption. The unadsorbed virus was removed and the cells were incubated. Viral titers were determined after three freeze–thaw cycles. The Δlog value was calculated relative to the viral controls (infected cells not treated with the extracts) for each corresponding time interval. Each sample was analyzed in four independent replicates, and the arithmetic mean value was determined (n = 4). In each replicate, every sample was represented by four individual wells in the cell culture plate.

### 3.14. Antiproliferative Activity

An MTT test [52] was used to evaluate antiproliferative activity. The assay was based on the metabolization of the tetrazolium salt MTT to formazan, whose concentration was determined photometrically. Measured optical density was used to assess cell viability. The cells (1 × 10^3^ cells/well in a 96-well plate) were incubated for 24 h in an incubator under standard conditions. Subsequently, the cells were incubated for 72 h with the test extracts at concentrations ranging from 4 to 1000 μg/mL. After washing twice with phosphate-buffered saline, a 0.5 mg/mL MTT solution was added (100 µL/well). The plates were incubated for 3 h, then washed with phosphate-buffered saline (pH 7.4), and 100 µL/well of lysis buffer (ethanol/DMSO = 1:1) was added. Extinction was measured at a wavelength of 540 nm.

Formula for calculating antiproliferative activity:

Antiproliferative activity (%) = (1 − (OD_540_ (sample)/OD_540_ (negative control)) × 100

### 3.15. Statistical Analysis

Statistical analysis was applied to process the data obtained from the cytotoxicity and antiviral effect experiments. The CC_50_ and IC_50_ were presented as mean values ± standard deviation from three independent replicates. The significance of the differences between the values for cytotoxicity and antiviral activity obtained from the experiments was determined by one-way ANOVA, followed by Bonferroni’s post hoc test by GraphPad Prism 8 software (San Diego, CA, USA). Statistical significance was accepted at *p* < 0.05. The effect obtained on virucidal activity was compared with the effect obtained with 70% ethanol. The effect on viral adsorption and the protective effect on cells were compared with the viral control at the corresponding time interval.

## 4. Conclusions

The presented study provides an in-depth description of the phenolic composition and the in vitro biological activities of methanolic and hydromethanolic extracts of *A. eupatoria*. Both extracts were rich in phenolic constituents, particularly flavonoid glycosides, flavan-3-ols, and hydrolysable tannins, with luteolin 7-*O*-glucoside, isoquercetin, hyperoside, and agrimoniin among the major compounds identified. Both extracts exhibited antiviral activity against HSV-1 and HCoV-OC43 at various stages of the virus–host interaction under in vitro conditions. The effects were slightly more pronounced against HSV-1. Both extracts exert a significantly stronger protective effect in Vero E6 cells. In addition, the extracts showed low cytotoxicity, no phototoxic potential under the experimental conditions, and selective antiproliferative activity against MCF-7 and MDA-MB-231 breast cancer cells compared with non-tumorigenic MCF-12F cells. Overall, these findings highlight *A. eupatoria* as a source of phenolic-rich extracts exhibiting in vitro antiviral and antiproliferative activities. Various biological properties have been reported for some of the identified constituents; however, their contribution to the effects we observed remains unknown at this stage. Further research is required to clarify their significance regarding biological activities and to gain a better understanding of the underlying mechanisms of their action.

## Figures and Tables

**Table 1:** Data from the analysis of *A. eupatoria* extracts.

Peak	Rt ^a^ (min)	Rt ^b^ (min)	Compound	Molecular Formula	[M−H]^−^	Δppm	Fragments	Ae-M, mg/g DE	Ae-WM, mg/g DE	Reference
1	0.91	3.66	Quinic acid ^c^	C_7_H_12_O_6_	191.0554	−3.54	191, 127, 85	nd	nd	
2	0.92	4.13	3-*O*-Caffeoyl-5-*O*-malonylquinic acid	C_19_H_20_O_12_	439.0885	0.62	439, 291, 155, 97, 79	nd	nd	[27]
3	0.95	13.77	Procyanidin dimer	C_30_H_26_O_12_	577.1360	1.56	577, 407, 299, 289, 245, 161, 125	0.94 ^e^	0.79	[28]
4	0.95	12.68	Chlorogenic acid isomer	C_16_H_18_O_9_	353.0883	1.45	353, 191, 179, 135	1.49 ^e^	1.24	
5	0.96	14.98	Procyanidin trimer	C_45_H_38_O_18_	865.1994	1.63	865, 695, 577, 449, 407, 287, 243, 161, 125	0.85 ^e^	0.66	[25]
6	0.99	12.26	Pedunculagin	C_34_H_24_O_22_	783.0707	2.67	783, 481, 301, 275, 257, 229	0.39 ^e^	0.34	[28]
7	1.53	12.70	Neochlorogenic acid ^c^	C_16_H_18_O_9_	353.0883	1.28	353, 191, 179, 135	1.49 ^e^	1.24	
8	2.23	15.90	(+)-Catechin ^c^	C_15_H_14_O_6_	289.0721	1.32	289, 245, 203, 179, 161, 151, 137, 123, 109	1.10 ^e^	0.91	
9	2.25	15.69	3-*O*-*p*-Coumaroylquinic acid	C_16_H_18_O_8_	337.0934	1.64	337, 191, 163, 119	2.50 ^e^	2.14	[28]
10	3.73	19.50	(−)-Epicatechin ^c^	C_15_H_14_O_6_	289.0722	1.64	289, 245, 203, 179, 161, 151, 137, 123, 109	2.17 ^e^	1.77	
11	7.22	24.92	Ellagic acid ^c^	C_14_H_6_O_8_	300.9994	1.33	301	1.45 ^e^	1.31	
12	7.98	25.28	Hyperoside (Quercetin 3-*O*-galactoside) ^c^	C_21_H_20_O_12_	463.0893	2.63	463, 301, 300, 271, 255, 243	10.36 ^f^	5.24	
13	8.10	24.43	Rutin (Quercetin 3-*O*-rutinoside) ^c^	C_27_H_30_O_16_	609.1465	0.62	609, 300, 271, 255, 243	0.36 ^f^	0.12	
14	8.45	25.67	Isoquercetin (Quercetin 3-*O*-glucoside) ^c^	C_21_H_20_O_12_	463.0889	1.51	463, 301, 300, 271, 255, 243	13.16 ^f^	5.30	
15	8.94	26.13	Luteolin 7-*O*-glucoside ^c^	C_21_H_20_O_11_	447.0944	2.41	447, 285	30.22 ^f^	9.55	
16	9.19	22.90	Agrimoniin ^d^	C_82_H_54_O_52_	934.0741	2.54	1085, 935, 783, 633, 301	7.63 ^e^	4.30	[25]
17	9.79	27.25	Quercetin malonylhexoside	C_24_H_22_O_15_	549.0895	1.72	505, 300, 271, 179	0.46 ^f^	0.23	[25]
18	10.16	27.07	Kaempferol 3-*O*-rutinoside ^c^	C_27_H_30_O_15_	593.1524	2.04	593, 285, 255, 227	2.18 ^f^	1.81	
19	10.58	28.51	Astragalin (Kaempferol 3-*O*-glucoside) ^c^	C_21_H_20_O_11_	447.0944	2.41	447, 284, 255, 221	2.73 ^f^	1.68	
20	10.71	29.45	Quercitrin (Quercetin 3-*O*-rhamnoside) ^c^	C_21_H_20_O_11_	447.0939	1.31	447, 301, 300, 271, 255, 243, 227	4.97 ^f^	1.89	
21	11.29	29.64	Apigenin 7-*O*-glucuronide ^c^	C_21_H_18_O_11_	445.0784	1.79	269	0.77 ^f^	0.68	
22	11.31	29.91	Apigenin 7-*O*-glucoside ^c^	C_21_H_20_O_10_	431.0990	1.46	431, 272, 268	nd	nd	
23	12.32	29.44	Kaempferol malonylhexoside	C_24_H_22_O_14_	533.0947	1.85	489, 285, 133	2.36 ^f^	1.18	[25]
24	15.75	36.84	Luteolin ^c^	C_15_H_10_O_6_	285.0408	1.19	285, 133	0.61 ^f^	0.47	
25	17.28	36.49	*trans*-Tiliroside (Kaempferol-3-*O*-(6”-*O*-*p*-coumaroyl)-glucoside) ^c^	C_30_H_26_O_13_	593.1312	1.86	593, 285, 255, 227	2.02 ^f^	4.18	
26	18.25	37.13	*cis*-Tiliroside (Kaempferol-3-*O*-(6”-*O*-*p*-coumaroyl)-glucoside)	C_30_H_26_O_13_	593.1312	1.86	593, 285, 255, 227	0.48 ^f^	1.04	[29]
27	22.15	39.56	Kaempferol 3-*O*-(2″-*O*-acetyl-6″-*p*-coumaroyl)-glucopyranoside I	C_32_H_28_O_14_	635.1420	2.11	635, 285, 255, 227	1.07 ^f^	2.65	[30]
28	22.90	39.66	Kaempferol 3-*O*-(2″-*O*-acetyl-6″-*p*-coumaroyl)-glucopyranoside II	C_32_H_28_O_14_	635.1418	1.82	635, 285, 255, 227	0.22 ^f^	0.68	[30]

^a^ Retention time HPLC-HRMS/MS; ^b^ Retention time HPLC-PDA-ESIMS; ^c^ reference standards; ^d^ [M−2H]^2−^; ^e^ Results are expressed as milligram ellagic acid equivalent in gram dry extract (mg EA/g DE); ^f^ Results are expressed as mg hyperoside equivalent in gram dry extract (mg HypE/g DE); nd—not determined.

**Table 2:** Cytotoxicityand phototoxicity of Ae-M and Ae-WM extracts, in BALB 3T3 cells, expressed as TC_50_ values ± SD and PIF.

Compounds	TC_50_ ± SD (μg/mL)		PIF *
	−Irr	+Irr **	
Ae-M	642.23 ± 31.16	598.42 ± 26.18	1.07
Ae-WM	1020.53 ± 70.93	704.02 ± 14.75	1.45
Chlorpromazine ***	11.72 ± 0.412	1.856 ± 0.317	6.31

* Photo Irritation Factor: PIF < 2 not phototoxic, 2 < PIF < 5 probable phototoxicity, PIF > 5 phototoxic, ** Irr—Irradiation, *** Chlorpromazine (positive control).

**Table 3:** Cytotoxicity and antiviral activity of the tested samples.

Compound Tested	Cytotoxicity (μg/mL)				Antiviral Activity (μg/mL)			
	MDBK		Vero E6		HSV-1		HCoV-OC43	
	CC_50_ Mean ± SD	MTC	CC_50_ Mean ± SD	MTC	IC_50_ Mean ± SD	SI	IC_50_ Mean ± SD	SI
Ae-M	687.4 ± 7.3 ***	250.0	658.1 ± 9.3 ***	100.0	48.1 ± 4.2 ***	14.3	88.5 ± 3.5 ***	7.4
Ae-WM	796.3 ± 8.8 ***	250.0	737.9 ± 9.5 ***	100.0	49.1 ± 3.6 ***	16.2	142.4 ± 5.2 ***	5.1
ACV	291.0 ± 9.4	nd	nd	nd	0.33 ± 0.03	881.8	nd	nd
REM	nd	nd	129.2 ± 9.3	nd	nd	nd	1.4 ± 0.3	92.3

CC_50_—cytotoxic concentrations 50%; SD—Standard deviation; MTC—maximum tolerable concentration; IC_50_—inhibitory concentration 50%; SI—selectivity index, which was calculated from the CC_50_/IC_50_ ratio; nd—no data; REM—Remdesivir; ACV—acyclovir; *** *p* < 0.001, when comparing the value of each sample with the corresponding reference substance; one-way ANOVA, followed by Bonferroni’s post hoc test; *n* = 3.

**Table 4:** Virucidal activity of the *A. eupatoria* extracts.

Viral Strain	Sample	Δlg				
		15 min	30 min	60 min	90 min	120 min
HSV-1	Ae-M	0.67	1.0	2.0	2.65	3.0
	Ae-WM	0.67	1.0	2.0	2.5	2.75
	70% ethanol	5.0	5.0	5.0	5.0	5.0
HCoV-OC43	Ae-M	2.33	2.33	2.33	2.33	2.33
	Ae-WM	1.75	1.75	1.75	2.0	2.0
	70% ethanol	5.0	5.0	5.0	5.0	5.0

**Table 5:** Influence of the *A. eupatoria* extracts on the viral adsorption stage.

Viral Strain	Sample	Δlg				
		15 min	30 min	45 min	60 min	120 min
HSV-1	Ae-M	4.0	4.5	5.0	5.0	
	Ae-WM	4.75	4.75	5.0	5.0	
HCoV-OC43	Ae-M	0.25	0.5	0.5	1.0	1.0
	Ae-WM	0.25	0.5	0.5	1.0	1.0

**Table 6:** Protective effect of *A. eupatoria* extracts on healthy cells.

Viral Strain	Sample	Δlg				
		15 min	30 min	60 min	90 min	120 min
HSV-1	Ae-M	0.5	1.0	1.5	1.75	1.75
	Ae-WM	1.25	1.5	1.5	1.75	1.75
HCoV-OC43	Ae-M	2.5	3.0	3.25	3.25	3.25
	Ae-WM	2.5	3.0	3.75	3.75	3.75

**Table 7:** Mean IC_50_ values and selectivity index (SI).

Extracts	Mean IC_50_ ± SD (µg/mL)			SI *	
	MCF-12F	MCF-7	MDA-MB-231	MCF-7	MDA-MB-231
Ae-M	296.94 ± 12.15	140.63 ± 7.9	93.3 ± 3.79	2.11	3.18
Ae-WM	383.23 ± 23.56	104.65 ± 4.68	115.41 ± 5.37	3.66	3.32
Cisplatin **	13.41 ± 1.41	22.52 ± 1.35	4.59 ± 0.23	0.59	2.92

* SI—Selectivity index, SI = IC_50_ normal cell line/IC_50_ tumor cell line, ** Cisplatin—(Positive control).

## Data Availability

The raw data supporting the conclusions of this article will be made available by the authors upon request.
